# MiR-148a impairs Ras/ERK signaling in B lymphocytes by targeting SOS proteins

**DOI:** 10.18632/oncotarget.17662

**Published:** 2017-05-07

**Authors:** Julia Alles, Nicole Ludwig, Stefanie Rheinheimer, Petra Leidinger, Friedrich A. Grässer, Andreas Keller, Eckart Meese

**Affiliations:** ^1^ Institute of Human Genetics, Saarland University, 66421 Homburg, Germany; ^2^ Institute of Virology, Saarland University, 66421 Homburg, Germany; ^3^ Chair for Clinical Bioinformatics, Saarland University, 66123 Saarbrücken, Germany

**Keywords:** B cells, microRNA, miR-148a, SOS, ERK

## Abstract

Although microRNAs have been recognized as central cellular regulators, there is an evident lack of knowledge about their targets. Here, we analyzed potential target genes for miR-148a functioning in Ras signaling in B cells, including SOS1 and SOS2. A dual-luciferase reporter assay showed significantly decreased luciferase activity upon ectopic overexpression of miR-148a in HEK-293T cells that were co-transfected with the 3′UTR of either SOS1 or SOS2. Each of the 3′UTRs of SOS1 and SOS2 contained two binding sites for miR-148a both of which were necessary for the decreased luciferase activity. MiR-148a overexpression in HEK-293T lead to significantly reduced levels of both endogenous SOS1 and SOS2 proteins. Likewise, reduced levels of SOS proteins were found in two B cell lines that were transfected with miR-148a. The level of ERK1/2 phosphorylation as one of the most relevant downstream members of the Ras/ERK signaling pathway was also reduced in cells with miR-148a overexpression. The data show that miR-148a impairs the Ras/ERK signaling pathway via SOS1 and SOS2 proteins in B cells.

## INTRODUCTION

Antigens binding to B cell receptors (= BCR) stimulate a signal cascade starting with a kinase cascade managing the activation of Ras proteins by guanine nucleotide exchange factors including SOS proteins. Active Ras-GTP subsequently activates the serine/threonine kinase Raf which phosphorylates and activates the protein kinases MEK1/2 which in turn phosphorylate and activate the extracellular signal-regulated protein kinases p44/p42 (ERK1/2) [1]. Ras/ERK signaling has a crucial impact on B lymphocyte development [2, 3], activation and survival [4–6]. In addition, Ras/ERK signaling plays an essential role in the malignant transformation of > 90% of all carcinomas (reviewed in [7, 8] ).

MiRNAs represent a class of non-coding nucleic acid molecules that are approximately 20 nucleotides in length. For *homo sapiens*, 2,500 different mature miRNAs are currently included in the database MiRBase [9]. Others and we have reported disease-specific miRNA expression profiles in whole peripheral blood. In detail, we identified miRNA profiles from whole peripheral blood of patients with myocardial infarction [10], lung cancer [11–13], multiple sclerosis [14, 15], melanoma [16], ovarian cancer [17], chronic obstructive pulmonary disease [18] and Alzheimer disease [19]. By analyzing more than 1,000 genome-wide miRNA profiles we identified 34 blood-borne miRNAs that are dysregulated in human pathologies [20]. While these studies clearly support the idea of miRNAs being crucial for regulating protein expression in blood cells, there are only two reports investigating human miRNA expression patterns in different blood cell subsets [21, 22]. Recently, we reported miRNA expression patterns in CD3+ T cells, CD19+ B cells, CD56+ NK cells, CD14+ monocytes and CD15+ eosinophilic and neutrophilic granulocytes in healthy individuals and patients suffering from lung carcinoma [23].

MiRNAs influence many cellular pathways by fine-tuning cellular protein expression. In total, more than 50% of all genes in the human genome are predicted to be miRNA targets [24]. The overwhelming majority of these predicted targets awaits, however, experimental confirmation [25]. Several comprehensive databases like TarBase [26] and miRTarBase [27] provide information on miRNA-target interactions. To identify predicted target genes that accumulate in certain KEGG pathways or gene ontologies, we developed a dictionary of miRNAs and their putative target pathways [28]. Recently, we reported miRNA Pathway Dictionary Database (miRPathDB), a freely accessible database (https://mpd.bioinf.uni-sb.de/) that summarizes information on pathways regulated by a miRNA, on miRNAs targeting a pathway and on the specificity of these regulations [29]. Based on our aforementioned genome-wide analyses of miRNAs in blood cell fractions, we identified miRNAs that can control the expression of an entire gene family. Specifically, members of the protein kinase C family (PKC) were targeted by miR-34a-5p that was significantly up-regulated in CD3+ peripheral blood cells of lung cancer patients [30].

In a previous study, we analyzed altered miRNA patterns in B cells of patients with lung cancer [23]. Here, we set out to answer the questions whether central signaling pathways of B cells are regulated by specific miR-148a-3p. In detail, we investigated key members of the BCR signaling cascade for their responsiveness to a regulation by miR-148a-3p. The members of the Ras/ERK signaling pathway that were analyzed for miRNA influence included SYK, GRB2, RasGRP3, NRAS, RAF1, MEK1, ERK2, FOS, SOS1, and SOS2. The cloned 3′UTR sequences of the respective mRNAs were used in a dual-luciferase reporter assay. Changes of the endogenous protein expressions as a result of ectopic miRNA expression were monitored in HEK-293T cells and in the B cell lines BJAB, DG-75 and U2932. In addition, the phosphorylation levels of ERK1/2 were also investigated as a potential downstream effect of an attenuated Ras activation.

## RESULTS

### miR-148a-3p target gene prediction and validation

**Figure 1:**
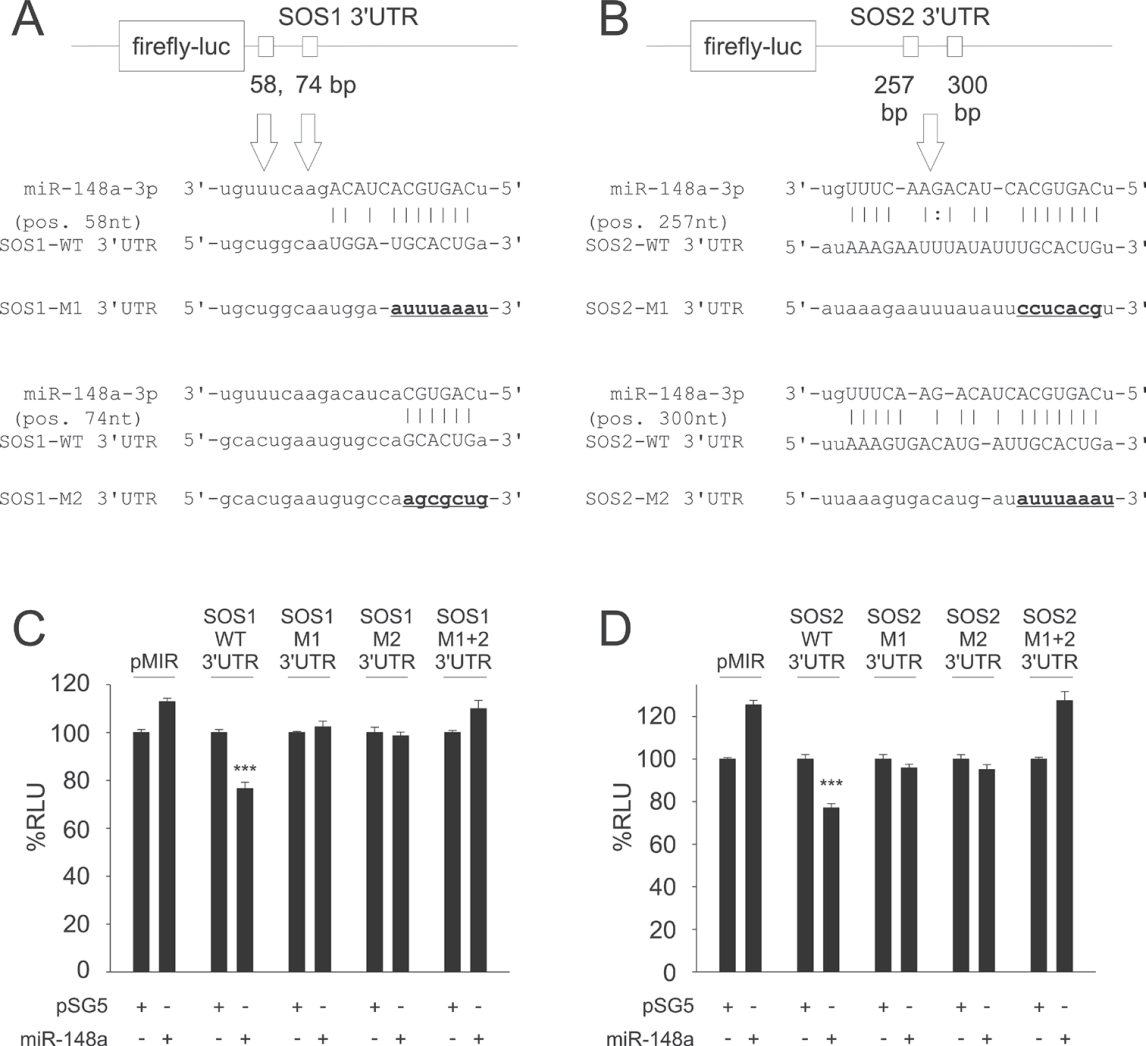
Predictions of potential binding sites for hsa-miR-148a-3p within the 3′UTRs of SOS1 (**A**) and SOS2 (**B**) and Dual-luciferase reporter assays for SOS1 (**C**) and SOS2 (**D**) as targets for miR-148a. Upper panels: Target site predictions. Annealing nucleotides between miR-148a-3p and its wildtype (WT) binding sites in SOS1 (A) and SOS2 (B) 3′UTRs are represented in upper case letters. Site directed mutagenesis of binding sites for miR-148a-3p (M1, M2) is indicated by underlined nucleotides in bold. Lower panels: Dual-luciferase assays. A pSG5 plasmid expressing miR-148a (or the empty pSG5 vector as a control) was co-transfected into HEK-293T cells either with the empty pMIR reporter vector, with pMIR reporter vector containing SOS1 (C) or SOS2 (D) wildtype (WT) 3′UTR, with SOS1 or SOS2 reporter vectors containing a single mutated binding site each (M1, M2) or with pMIR reporter vector for SOS1 (C) or SOS2 (D) containing both mutated binding sites (M1+2) each for miR-148a-3p. Co-transfection of miR-148a with wildtype 3′UTR reporter vectors resulted in relative light units significantly reduced to 77% for both SOS1 (C) or SOS2 (D) reporters as compared to control samples (*P* < 0.001 each). The downregulation of luciferase activity was abrogated as soon as at least one of the two miR-148a-3p binding sites was mutated.

Previously, we reported miRNA expression patterns in different blood cell subtypes in lung carcinoma patients and healthy individuals [23]. The abundance of miR-148a-3p was significantly up-regulated in CD19+ B cells (*P* = 0.03) with a 2.47-fold change in lung cancer patients as compared to healthy individuals (Table 1). MiR-148a was the only miRNA that was significantly deregulated in B cells but not in any other lymphocyte subpopulation analyzed. Other miRNAs, for example miR-34a and miR-144, that were likewise deregulated in B cells, were also deregulated in CD3+ cells and CD15+ cells, respectively (Supplementary Table 1). Target prediction for miR-148a-3p by *in silico* analyses using miRWalk 2.0 [31] yielded 47,317 potential target genes. By limiting our analysis to genes, which were predicted by at least 5 out of 11 algorithms, we obtained 6,557 potential target genes for miR-148a-3p. Among these predicted targets, we identified miR-148a-3p binding sites within the 3′UTRs of SOS1 (Son of Sevenless 1; predicted by 6 algorithms, 2 binding sites) and SOS2 (Son of Sevenless 2; predicted by 8 algorithms, 2 binding sites), as shown in Figure 1. Since both genes function in Ras signaling, we also included other genes of this particular pathway in our study. In detail, we found potential binding sites for miR-148a-3p within the 3′UTRs of SYK, GRB2, RasGRP3, NRAS, RAF1, MEK1, ERK2 and FOS (Supplementary Figure 1).

**Table 1:** Differentially expressed miRNAs in CD19+ peripheral blood cells from lung cancer patients vs. healthy individuals

CD19+ lymphocytes	median healthy donors	median LCa patients	ratio L/H	FC	*P*
hsa-miR-34a-5p	3.46	4.86	1.41	2.64	0.009
hsa-miR-148a-3p	4.63	5.93	1.28	2.47	0.031
hsa-miR-144-3p	5.67	3.60	0.63	0.24	0.003

Log-transformed normalized expression data originate from Leidinger et al., 2014 [23], LCa: lung cancer, FC: fold change, *P*: *P*-value.

**Figure 2:**
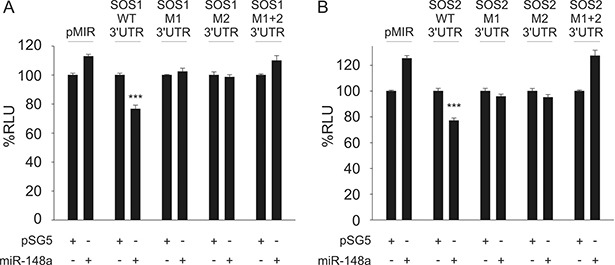
Dual-luciferase reporter assays for SOS1 (**A**) and SOS2 (**B**) as targets for miR-148a. A pSG5 plasmid expressing miR-148a (or the empty pSG5 vector as a control) was co-transfected into HEK-293T cells either with the empty pMIR reporter vector, with pMIR reporter vector containing SOS1 (A) or SOS2 (B) wildtype (WT) 3′UTR, with SOS1 or SOS2 reporter vectors containing a single mutated binding site each (M1, M2) or with pMIR reporter vector for SOS1 (A) or SOS2 (B) containing both mutated binding sites (M1+2) each for miR-148a-3p. Co-transfection of miR-148a with wildtype 3′UTR reporter vectors resulted in relative light units significantly reduced to 77% for both SOS1 (A) or SOS2 (B) reporters as compared to control samples (*P* < 0.001 each). The downregulation of luciferase activity was abrogated as soon as at least one of the two miR-148a-3p binding sites was mutated.

For validation of the potential targets, we first cloned the sequences of the predicted 3′UTR targets into the pMIR-RNLTK dual-luciferase reporter vector and co-transfected the reporter with the miR-148a expression vector into HEK-293T cells. The ectopic overexpression of miR-148a in HEK-293T cells that were co-transfected with the 3′UTR SOS1 reporter vector lead to a significant decrease of the luciferase activity (23%). Likewise, the co-transfection of miR-148a with the 3′UTR SOS2 reporter also lead to a 23% reduction of the luciferase activity (Figure 2). To control for off-target effects, we mutated both miR-148a-3p binding sites within 3′UTRs both of SOS1 and SOS2. Co-transfection of miR-148a with either of the two mutated 3′UTRs of SOS1 did not cause a decrease of the luciferase activities at all. These results indicate that both binding sites for miR-148a within the 3′UTRs of SOS1 are necessary to mediate the biological effect of the miR-148a binding. As expected from these results, co-transfection of miR-148a with both mutated 3′UTRs combined also failed to reduce the luciferase activity (Figure 2A). Similar results were obtained for SOS2. We co-transfected miR-148a with each of the two mutated 3′UTRs of SOS2 separately and with both mutated 3′UTRs combined. None of the transfections with the mutated 3′UTRs yielded a decreased luciferase activity indicating that both binding sites within the 3′UTRs of SOS2 are necessary to mediate the biological effect of the miR-148a binding (Figure 2B). These results confirm SOS1 and SOS2 as direct targets of miR-148a-3p and that for both targets, both miRNA binding sites are needed to exert the inhibitory function of miR-148a-3p.

As indicated above, other members of the Ras signaling pathway also included genes with potential binding sites for miR-148a-3p. For each of these members, we cloned the sequences of particular target mRNAs into the pMIR-RNLTK dual-luciferase reporter vector and co-transfected the reporter with a miR-148a expression vector into HEK-293T cells. In detail, we analyzed the potential binding sites for miR-148a-3p within the 3′UTRs of SYK, GRB2, RasGRP3, NRAS, RAF1, MEK1, ERK2 and FOS. The dual-luciferase reporter assays did not show a decrease in luciferase activity for any of these potential miR-148a targets, as indicated in Supplementary Figure 2.

### Effect of miR-148a-3p expression on SOS protein levels

**Figure 3:**
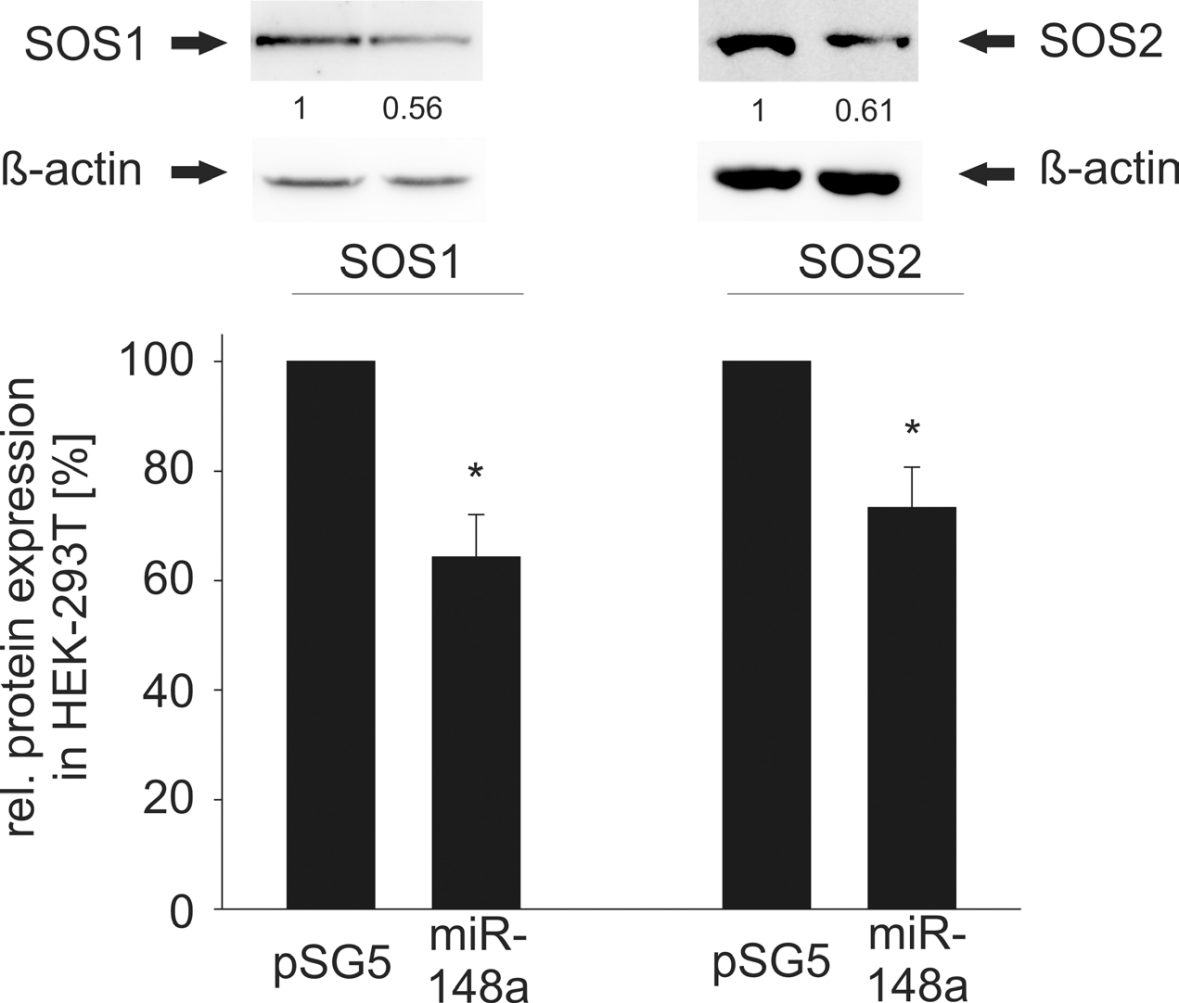
Western Blot analysis of miR-148a effects on endogenous SOS1/2 protein levels in HEK-293T cells Ectopic overexpression of miR-148a results in significantly decreased protein levels of SOS1 (**A**) and SOS2 (**B**). In detail, SOS1 expression was reduced to 64% (*P* = 0.01) and SOS2 to 73% (*P* = 0.02) in HEK-293T cells.

**Figure 4:**
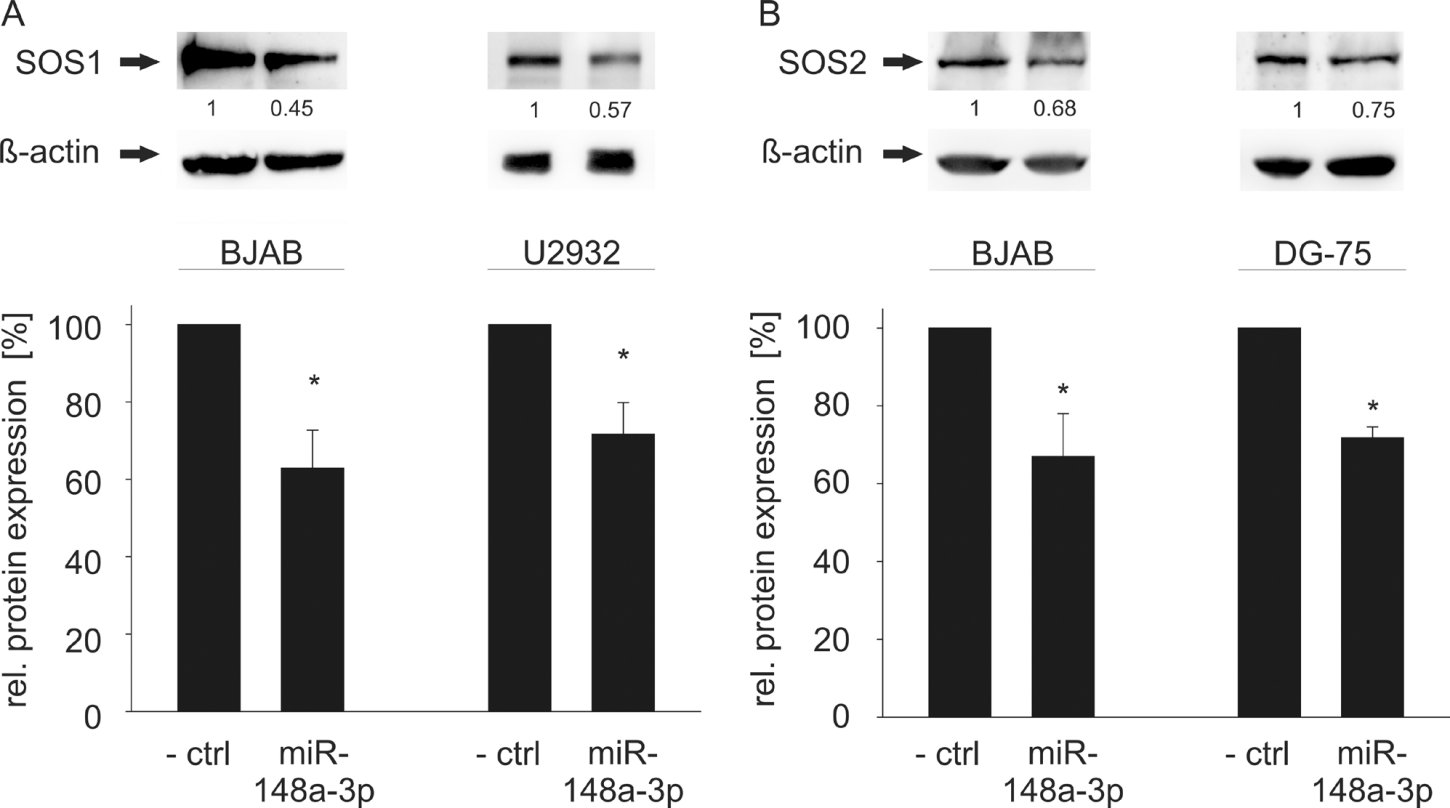
Western Blot analysis of miR-148a-3p effects on endogenous SOS1/2 protein levels in B cell lines (**A**) Transfection of miR-148a-3p using miRNA mimics causes significantly reduced SOS1 expression in BJAB (63%, *P* = 0.009) and U2932 (72%, *P* = 0.026) cells. (**B**) Transfection of miR-148a-3p mimics results in significantly decreased SOS2 protein levels in BJAB (67%, *P* = 0.04) and DG-75 (72%, *P* = 0.0006) cells. The expression of SOS1 in DG-75 and SOS2 in U2932 cells was considered as too low for further downregulation, as shown in Supplementary Figure 3.

To confirm the regulatory function of miR-148a-3p on protein level, we tested its effect on the endogenous protein level in different cell lines. To this end, we transfected HEK-293T cells with the miR-148a expression vector and measured the endogenous SOS1 and SOS2 proteins. As a consequence of the miR-148a overexpression, endogenous protein levels of SOS1 and SOS2 significantly decreased to 64% and 73%, respectively (*P* = 0.01 for SOS1 and *P* = 0.02 for SOS2) (Figure 3). Likewise, we analyzed the endogenous protein levels in the three B cell lines BJAB, DG-75 and U2932 by transfection with miR-148a-3p mimics by Western Blotting (Figure 4). In untransfected B cell lines, we measured different amounts of SOS1 and SOS2 protein. The expression of SOS1 in DG-75 cells was very low and SOS2 expression in U2932 cells was not detectable by Western Blotting (Supplementary Figure 3). Thus, the effect of miR-148a on SOS target genes is demonstrated in two B cell lines. As depicted in Figure 4, transfection of miR-148a-3p mimics also reduces endogenous SOS1 expression to 63% in BJAB and to 72% in U2932 cells (*P* = 0.009 for SOS1 in BJAB and 0.026 for SOS1 in U2932). SOS2 protein expression was decreased to 67% in BJAB and to 72% in DG-75 cells (*P* = 0.04 for SOS2 in BJAB and 0.0006 for SOS2 in DG-75) when transfected with miR-148a-3p mimics compared to cells transfected with “All Stars negative control”.

### Overexpression of miR-148a-3p inhibits ERK-activation

**Figure 5:**
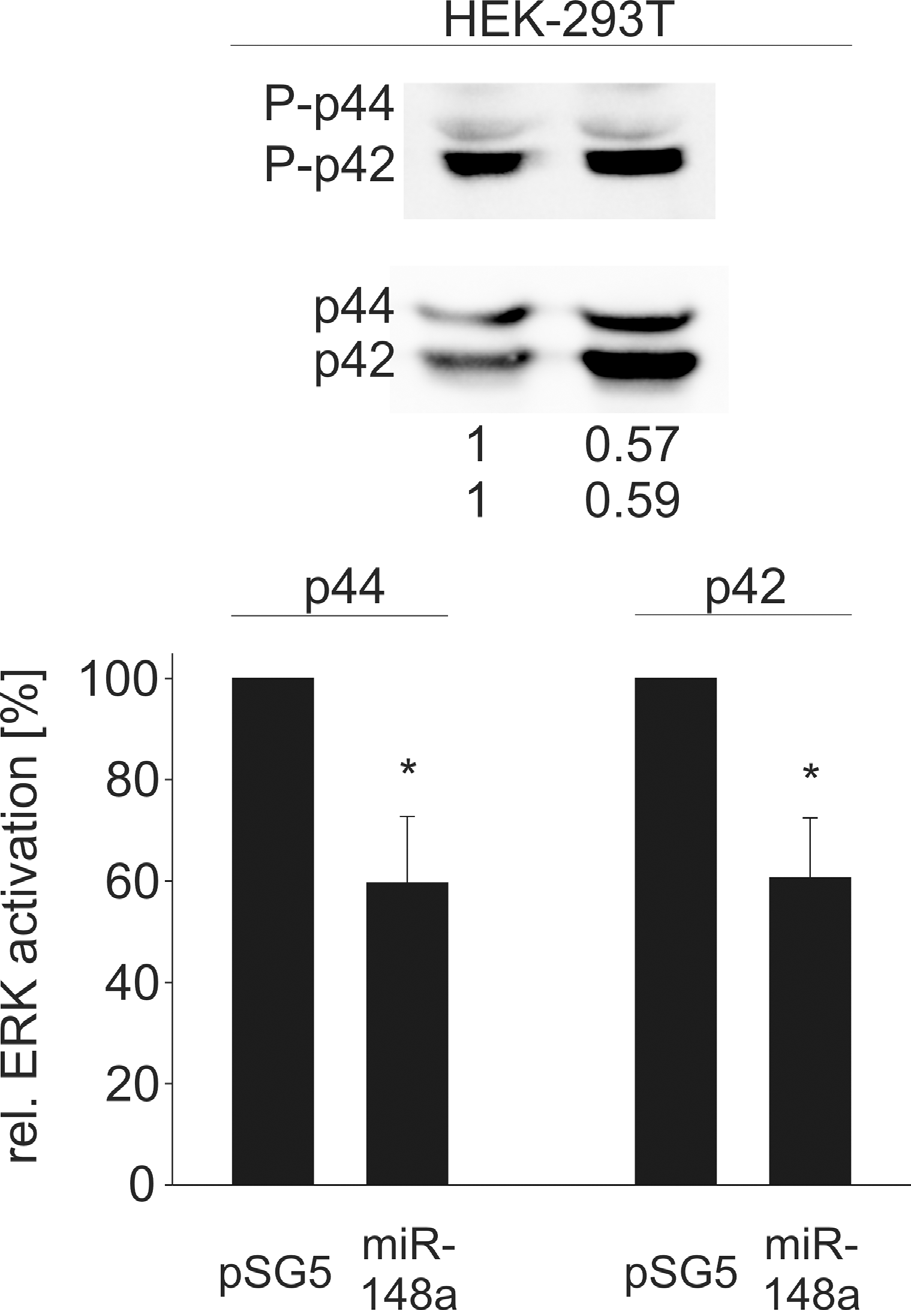
Western Blot analysis of miR-148a effects on the activation of ERK1/2 in HEK-293T cells Measurement of activated (phosphorylated) ERK1/2 as a downstream effect of decreased SOS1/2 protein levels due to overexpression of miR-148a results in significantly reduced phosphorylation of ERK1/p44 to 60% and ERK2/p42 to 61% (*P* = 0.02 each).

**Figure 6:**
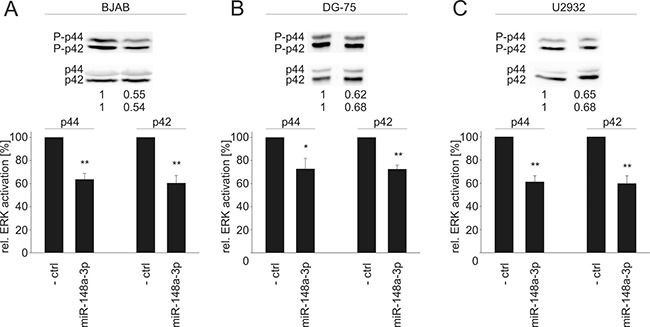
Western Blot analysis of miR-148a-3p effects on the activation of ERK1/2 in B cell lines A downstream effect of reduced SOS1/2 protein levels due to transfection of miR-148a-3p mimics causes significantly decreased activation (phosphorylation) of ERK1/2 to 64%/60% (*P* = 0.002/0.004) in BJAB cells (**A**), to 73% each (*P* = 0.04/0.001) in DG-75 cells (**B**), and to 61%/60/ (*P* = 0.002/0.004) in U2932 cells (**C**).

Since all tested novel target genes in our study are important elements in Ras signaling, we further investigated downstream effects of an elevated miR-148a-3p level by measuring the activation of ERK1/2, which is one of the central downstream targets of the Ras signaling pathway. Ectopic miR-148a-3p expression in HEK-293T cells affected ERK1 (p44) and ERK2 (p42) activation significantly (Figure 5). In detail, p44 and p42 phosphorylation was reduced to 60% and 61%, respectively (*P* = 0.02 for both p44 and p42). Similar results were achieved for three B cell lines (Figure 6). In BJAB cells, p44 phosphorylation was decreased to 64% and p42 phosphorylation was decreased to 60%, respectively (*P* = 0.002 and 0.004). In DG-75 cells, p44/p42 activation was diminished to 73% each (*P* = 0.04 and 0.001) and in U2932 cells p44/p42 phosphorylation was reduced to 61% and 60%, respectively (*P* = 0.002 and 0.004).

These results confirm a direct regulation of both SOS1 and SOS2 by miR-148a-3p in B cells resulting in an inhibition of the Ras/ERK signaling pathway demonstrated by reduced levels of ERK1/2 phosphorylation.

## DISCUSSION

In this study, we identified miR-148a as a central element in the regulation of Ras signaling. MiR-148a, that was previously associated with cancer development, exhibits both oncogenic and tumor suppressive features, and has been suggested as a biomarker for lung cancer [32–35]. In a recent study, we provided first evidence for upregulation of miR-148a in CD19+ B cells of lung cancer patients as compared to those isolated from healthy individuals [23].

Here, we identified the two Son of Sevenless members, SOS1 and SOS2, as direct targets for miR-148a-3p. SOS1 and SOS2 are human homologues for Son of Sevenless, which was originally discovered in *Drosophila melanogaster* [36, 37]. Besides being members of the same protein family, SOS1 and SOS2 share the same function and a rather similar ORF but with distinct differences in their 3′UTRs. In detail, the proteins have 70% amino acid homology, but no similarity between their 3′UTRs, which are 4.3 kb in length in case of SOS1 and 1.2 kb in case of SOS2. They function as Ras guanine nucleotide exchange factors by facilitating the conversion of the inactive Ras-GDP to the active GTP form [38].

Ras signaling plays an essential role in cell differentiation and growth. Active Ras-GTP subsequently activates the serine/threonine kinase Raf, which phosphorylates/activates the protein kinases MEK1/2, which in turn phosphorylate/activate the extracellular signal-regulated protein kinases p44/p42 (ERK1/2) [1]. In general, tyrosine kinase receptors are initial activators for Ras activation by SOS. Moreover, Ras/ERK signaling is required for B cell receptor mediated signal transduction, which plays important roles in the development, activation and survival of B lymphocytes [5, 6]. For immature B cells, BCR-induced Ras/ERK signaling results in growth arrest and apoptosis, whereas cell-cycle entry and activation occur after Ras activation in mature B cells [39, 40].

Similar to our findings in CD19+ B cells, the knockdown of SOS1/2 in CD4+ T cells results in decreased ERK phosphorylation upon T cell receptor (=TCR) activation [41]. In addition, SOS1/2 knock-out T cells show different migration behavior [42]. However, TCR-mediated ERK activation does not require SOS1/2 [43]. Therefore, the mechanisms of ERK1/2 activation seem to be different in B and T cells.

The binding of miR-148a to the 3′UTRs of either SOS1 or SOS2 lead to a similar reduction in luciferase activity indicating that the translation of both genes is largely similar affected by miR-148a binding. These results indicate that miR-148a inhibits SOS function. In accordance with this observation is our finding that both binding sites for miR-148a within the 3′UTRs of SOS1 contribute to the biological effect of the miR-148a binding. Likewise, both binding sites within the 3′UTRs of SOS2 also contribute to mediate the effect of miR-148a. Taken together, the overall effect of the miR-148a binding on the SOS activity appears to be mediated by two different binding sites in both SOS genes and in that may only be affected if one of the binding site is not functioning.

Besides SOS1 and SOS2 we found no evidence that other predicted target genes in the Ras/ERK signaling pathway are regulated by miR-148a. Nevertheless, miR-148a affects the entire pathway because the level of ERK1/2 activation is significantly reduced by an ectopic miR-148a expression.

Although our study shows that elevated miR-148a levels affect the Ras/ERK pathway in B cells, an impaired Ras-dependent BCR signaling does not affect the generation of IgG germinal center B cells. The recruitment of high-affinity cells into the memory compartment and the terminal differentiation are, however, inhibited by an impaired Ras-dependent BCR signaling [46]. As for plasma cells, ERK induces the expression of *Prdm1*, which encodes Blimp-1, the main transcriptional regulator of plasma cell differentiation [47–50]. B cells that are deficient for ERK1 and ERK2 show impaired Prdm1 expression, which results in an impaired generation of plasma cells. The generation of plasma cells can be restored by enforced expression of Prdm1 in the ERK-deficient B cells [47].

Interestingly, it was shown that miR-148a is the most abundant miRNA in mature plasma cells, and premature miR-148a expression favors plasmablast differentiation and survival of primary murine B cells by targeting of the Blimp-1 and IRF4 repressors Mitf and Bach2 [51]. It remains to been seen if and to what extend the translational inhibition of miR-148a on SOS1 and SOS2 genes and reduced ERK1/2 activity plays a role in plasma cell differentiation.

Likely, miR-148a is central to the signaling that occurs as result of B cell activation. In detail, BCR activation induces signaling of NFkB [52, 53], which in turn induces miR-148a [54]. MiR-148a targets RelA [55], which is instrumental for the formation of the NFkB-multimer. The exact role of miR-148a in this scenario, however, requires experimental evaluation.

While our study did not analyze alterations in B cell subsets, there is evidence for changes in lymphocytes in lung cancer patients under treatment. These changes include T cells and NK cells, but not B cells [56]. Germain and colleagues reported 50% less naive B cells and 50% more memory B cells in NSCLC patients versus controls [57]. A higher expression of miR-148a has been reported in memory B cells compared to naïve B cells separated from hyperplastic tonsil tissue [58]. Possibly, reported expression changes for miR-148a are influenced by changes of the ratio of naïve and memory B cells in lung cancer patients.

Besides its role in the Ras/ERK signaling pathway, miR-148a has multiple other targets many of which are related to tumor development. In lung cancer, miR-148a targets MMP15 and ROCK1, resulting in reduced tumorigenesis and increased TRAIL dependent apoptosis [34]. The regulation of STAT3 by miR-148a inhibits proliferation and invasion [59]. Among the various other targets of miR-148a are the cell cycle inhibitor CDKN1B (p27) and the tumor suppressor PTEN [60, 61]. The few as of now validated targets presumably represent only a small fraction of the complex targeting network of miR-148a.

Taken together, our data show that the Ras/ERK signaling pathway in B cells is regulated by miR-148a as shown for different cell culture systems including HEK-293T cells and B cell lines. Although other key members of the Ras signaling cascade, including SYK, GRB2, RasGRP3, NRAS, RAF1, MEK1, ERK2 and FOS are not directly regulated by miR-148a, we show that an elevated abundance of miR-148a affects the entire Ras/ERK signaling pathway ending in significantly reduced phosphorylation levels of endogenous ERK.

## MATERIALS AND METHODS

### Cell culture

HEK-293T cells were cultivated in DMEM (Life Technologies, Darmstadt, Germany), supplemented with 10% FBS (Biochrom, Berlin, Germany), 100 U/ml Penicillin and 100 μg/ml Streptomycin (Life Technologies, Darmstadt, Germany). The B cell lines BJAB and DG-75, both human Burkitt lymphoma cell lines [62, 63], and U2932, a diffuse large B cell lymphoma cell line [64], were grown in RPMI 1640 (Life Technologies, Darmstadt, Germany), supplemented with 10% FBS and 100 U/ml Penicillin and 100 μg/ml Streptomycin. All cell lines were previously described [30, 65–67].

### Dual luciferase reporter assays

Dual luciferase assays using different 3′UTR reporter vectors with pMIR-RNLTK backbone were carried out as described previously [30]. In brief, 1 × 10^5^ HEK-293T cells were seeded in 24-well cell culture plates. The next day, the cells were transfected using PolyFect (Qiagen, Hilden, Germany). After two more days, the assays were conducted according to dual-luciferase reporter assay system’s protocol (Promega, Mannheim, Germany). As controls for the pMIR-RNLTK dual-luciferase reporter, we used empty pMIR vector and for the miR-148a expression vector an empty pSG5 vector.

### Western blot

For Western Blotting, 4×10^5^ HEK-293T cells were seeded in 6-well cell culture plates. The next day, the cells were transfected with 2μg of pSG5-miR-148a expression plasmid or the empty pSG5 vector as a control using PolyFect (Qiagen, Hilden, Germany) according to the manufacturer’s protocol. 2 × 10^6^ B cells were seeded out in 6-well cell culture plates and subsequently transfected with 750ng hsa-miR-148a-3p miScript miRNA mimic (MIMAT0000243: 5′-UCA GUG CAC UAC AGA ACU UUG U-3′) or AllStars Negative Control (“- ctrl“) using HiPerFect (Qiagen, Hilden, Germany) following the manufacturer’s protocol. Six hours post transfection, the B cells were transferred to 25 cm^2^ cell culture flasks in a total of 10ml RPMI, supplemented with 10% FBS and 100 U/ml Penicillin and 100 μg/ml Streptomycin. 48 hours post transfection, the cells were harvested, washed twice with PBS and lysed using 2x protein sample buffer (130 mM Tris/HCl, 6% SDS, 10% 3-Mercapto-1,2-propandiol, 10% glycerol) following sonication. For 8% (SOS1/2) and 12% (pERK/ERK) SDS-PAGE, a total of 30 μg protein was separated and blotted onto nitrocellulose (GE Healthcare, Freiburg, Germany). The following primary antibodies were used: anti-SOS1 and anti-SOS2 (Santa Cruz Biotechnology, Heidelberg, Germany), anti-beta-actin (Sigma-Aldrich, Munich, Germany), anti-ERK1/2 and anti –phosphoERK1/2 (Cell Signaling Technology, Danvers, USA). The blots were developed using horseradish-coupled secondary antibodies (Sigma-Aldrich, Munich, Germany) and SignalFire ECL (Cell Signaling Technology, Danvers, USA) on a Chemidoc-Touch Imaging System (Bio-Rad, Munich, Germany).

### Plasmids

The pSG5-miR-148a expression vector was described previously [68]. For the generation of reporter plasmids, the 3′UTRs of the respective target genes were PCR-amplified from B cell cDNA and cloned into pMIR-RNLTK using unique restriction sites. Specific primer and restriction sites were listed in Supplementary Table 2. Mutation of miR-148a-3p binding sites was carried out using overlap extension PCRs.

### Data analysis

Statistical analysis of produced data was performed by SigmaPlot 10 software (Systat, Chicago, USA) using student’s *t-test*. The dual-luciferase reporter assays were performed at least three times in duplicate. Quantification of endogenous protein expression by Western Blotting was carried out using Image Lab software (Bio-Rad, Munich, Germany) on at least 3 independent protein extracts for miR-148a and controls.

## SUPPLEMENTARY MATERIALS FIGURES AND TABLES



## Abbreviations

ctrl: AllStars Negative Control
BCR: B cell receptor
CD: cluster of differentiation
DMEM: Dulbecco’s Modified Eagle Medium
ECL: enhanced chemiluminescence
FBS: fetal bovine serum
HEK: human embryonic kidney
LCa: lung cancer
miRNA: micro ribonucleic acid
ORF: open reading frame
NSCLC: non-small cell lung cancer
PAGE: polyacrylamide gel electrophoresis
PBS: phosphate-buffered saline
rel.: relative
TCR: T cell receptor
UTR: untranslated region
WT: wildtype
