# Supplementary material for: MiR-148a impairs Ras/ERK signaling in B lymphocytes by targeting SOS proteins
Source: Oncotarget. 2017 May 7;8(34):56417–27. doi: 10.18632/oncotarget.17662 (PMC5593572; doi:10.18632/oncotarget.17662)
Supplement: Supplementary file 1 [file oncotarget-08-56417-s001.pdf]

MiR-148a impairs Ras/ERK signaling in B lymphocytes by targeting SOS proteins

Supplementary Materials

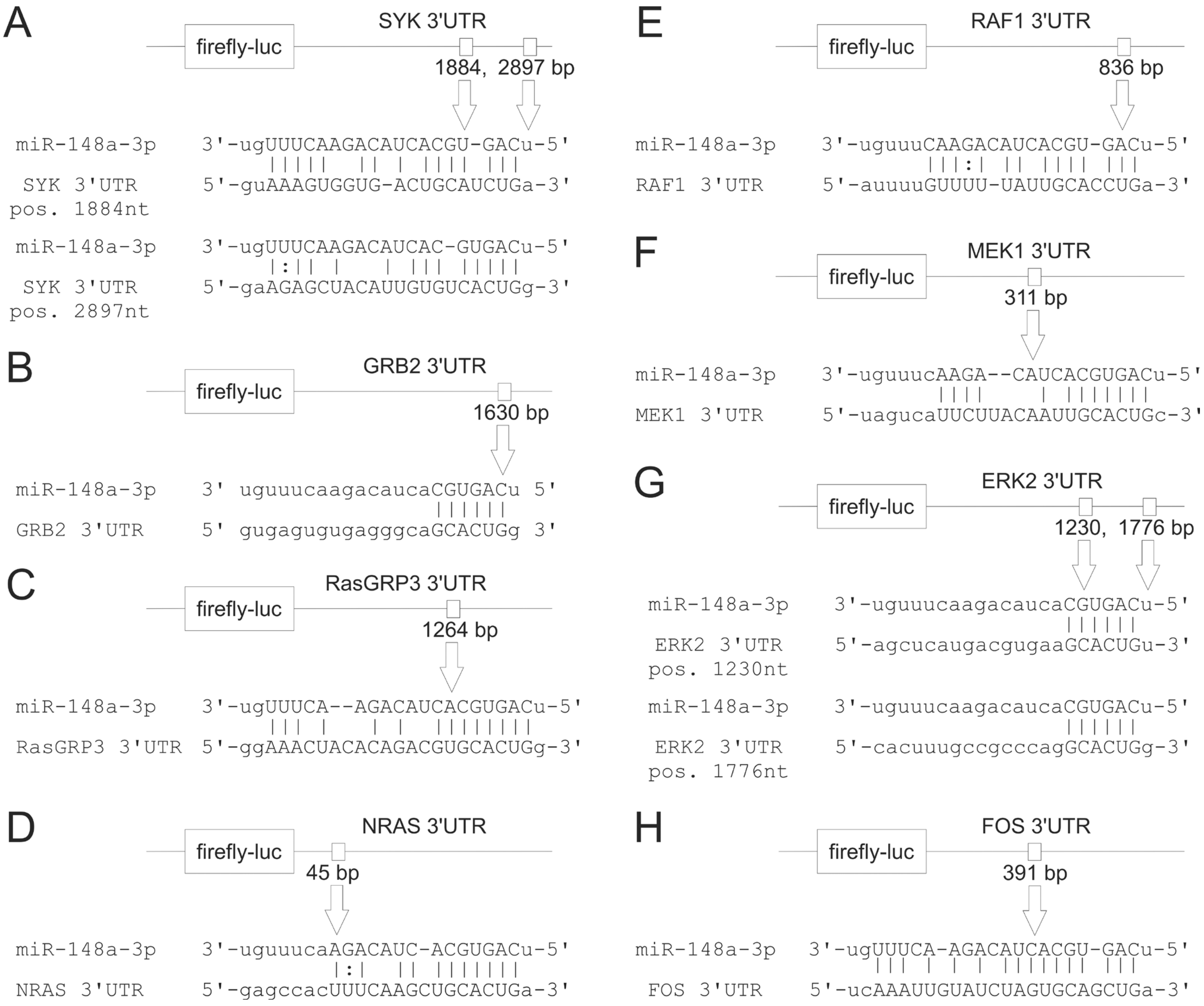

Supplementary Figure 1: Predictions of miR-148a-3p binding sites for Ras/ERK signaling members in B cells. The 3′UTRs of potential target genes SYK, GRB2, RasGRP3, NRAS, RAF1, MEK1, ERK2 and FOS contain up to two binding sites for miR-148a-3p. Annealing nucleotides are indicated in upper case letters.

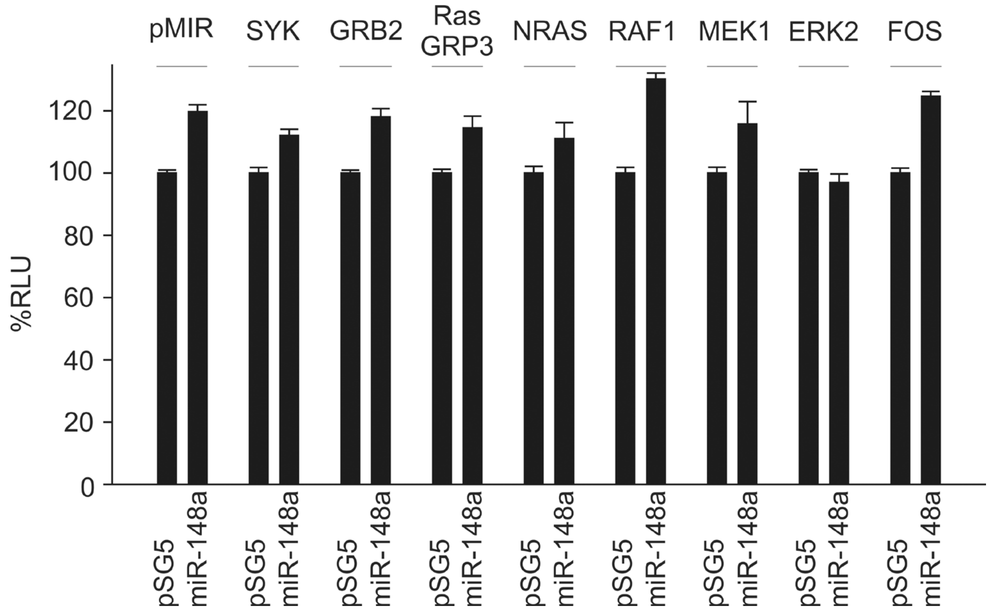

Supplementary Figure 2: Dual-luciferase reporter assays for Ras/ERK signaling members in B cells. For none of the analyzed potential targets a miR-148a dependent reduction of luciferase activity was detectable.

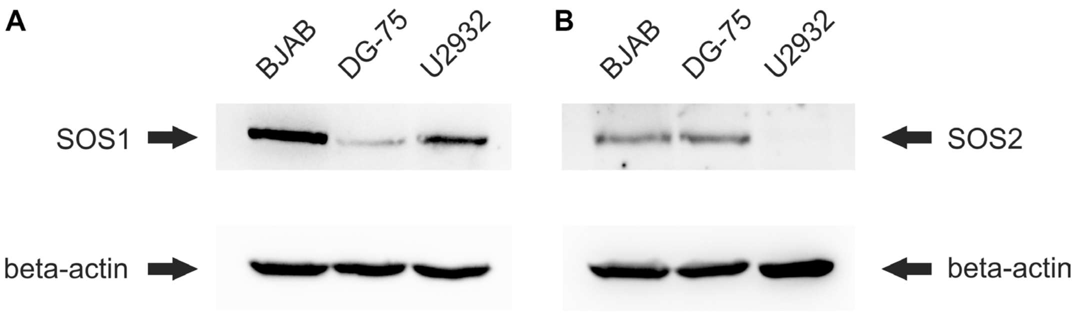

Supplementary Figure 3: Detection of endogenous SOS1 and SOS2 base levels in B cell lines by Western Blotting. Untreated BJAB, DG-75 and U2932 cells were used for the determination of base levels for endogenous SOS1 (**A**) and SOS2 (**B**) protein. (A) For transfection with miR-148a-3p mimic and the analysis of its down-regulating effect on SOS1 only the cell lines BJAB and U2932 were considered appropriate as the SOS1 protein level in DG-75 cells is rather low. (B) SOS2 is almost not detectable in U2932 cells by reason of which only BJAB and DG-75 cells were chosen for further experiments.

Supplementary Table 1: Mean values of in CD19+ cells from LCa patients vs. healthy individuals differentially expressed miRNAs in other separated blood cell populations

**CD19$^+$**	**median healthy donors**	**median LCa patients**	**ratio L/H**	**FC**	***P***
hsa-miR-34a-5p	3.46	4.86	1.41	2.64	0.009
hsa-miR-148a-3p	4.63	5.93	1.28	2.47	0.031
hsa-miR-144-3p	5.67	3.60	0.63	0.24	0.003
CD3$^+$					
hsa-miR-34a-5p	3.98	5.45	1.37	2.78	0.004
hsa-miR-148a-3p	6.06	5.79	0.96	0.83	0.138
hsa-miR-144-3p	3.62	2.51	0.69	0.46	0.167
CD14$^+$					
hsa-miR-34a-5p	3.35	3.77	1.12	1.33	0.238
hsa-miR-148a-3p	6.00	6.46	1.08	1.38	0.373
hsa-miR-144-3p	2.05	1.82	0.89	0.86	0.480
CD15$^+$					
hsa-miR-34a-5p	3.29	3.56	1.08	1.20	0.236
hsa-miR-148a-3p	8.71	8.99	1.03	1.21	0.365
hsa-miR-144-3p	4.70	3.18	0.68	0.35	0.042
CD56$^+$					
hsa-miR-34a-5p	3.83	4.63	1.21	1.74	0.169
hsa-miR-148a-3p	5.25	5.45	1.04	1.15	0.448
hsa-miR-144-3p	3.78	3.57	0.94	0.86	0.795

Log-transformed normalized expression data originate from Leidinger et al. (2014) [23], LCa: lung cancer, FC: fold change, *P*: *P*-value.

Supplementary Table 2: Oligonucleotides used for cloning of the reporter vectors analyzed in this study

Oligo name	sequence 5′—3′	restriction site
F-SOS1	gactagtcggaccttcattccattgctg	SpeI
R-SOS1	cgagctcggctgattactcaactatgtg	SacI
F-SOS2	gactagtcgtacagactgcctttgctag	SpeI
R-SOS2	cgagctcgcactctatcactttgaacg	SacI
F-SOS1-m1	cattgctggcaatggaatttaaatatgtgccagcactgag	SwaI
R-SOS1-m1	ctcagtgctggcacatatttaaattccattgccagcaatg	SwaI
F-SOS1-m2	gcactgaatgtgccaagcgctgggagttaaaatgag	AfeI
R-SOS1-m2	ctcattttaactcccagcgcttggcacattcagtgc	AfeI
F-SOS1-m1m2	cattgctggcaatggaatttaaatatgtgccaagcgctgggagttaaaatgagaac	SwaI, AfeI
R-SOS1-m1m2	gttctcattttaactcccagcgcttggcacatatttaaattccattgccagcaatg	SwaI, AfeI
F-SOS2-m1	tataaagaatttatattcctcagctaaactctgcaaaaatatg	BbvCI
R-SOS2-m1	catatttttgcagagtttagctgaggaatataaattctttatac	BbvCI
F-SOS2-m2	gtttaaagtgacatgatatttaaataaagggatagtgcttttg	SwaI
R-SOS2-m2	caaaagcactatccctttatttaaatatcatgtcactttaaac	SwaI
F-NRAS	gactagtcgatgtacctatggtgctagtg	SpeI
R-NRAS	gccggcgtataggtagtaacctatg	NgoMIV
F-Raf1	gactagtcggctggtagctgactgtgtg	SpeI
R-Raf1	cgagctcggagggaccatcagataactg	SacI
F-MEK1	gactagtccgtctaagtgtttgggaagc	SpeI
R-MEK1	cgagctcggagacataccactaagtatc	SacI
F-SYK	gactagtcggacagccatggacagcagg	SpeI
R-SYK	gccggcgtccagtgacacaatgtagc	NgoMIV
F-FOS	gactagtcgcagcagcaatgagccttcc	SpeI
R-FOS	cgagctcgcagaacacactattgccagg	SacI
F-RasGRP3	cgagctcggttatctggaaagatacctg	SacI
R-RasGRP3	gccggcggaatatatacactttagcc	NgoMIV
F-ERK2	gactagtccatgacgtgaagcactgttc	SpeI
R-ERK2	gccggcctgaatggctgaaatattac	NgoMIV
F-GRB2	gactagtcgacagagcgagattccgtctc	SpeI
R-GRB2	cgagctcgcttagttcagcaaggcttc	SacI
